# Patient, primary care provider, and stakeholder perspectives on mammography screening frequency: lessons learned from a qualitative study

**DOI:** 10.1186/s12885-022-09900-x

**Published:** 2022-07-27

**Authors:** Vicky Ro, Tarsha Jones, Thomas Silverman, Julia E. McGuinness, Ashlee Guzman, Jacquelyn Amenta, Rita Kukafka, Katherine D. Crew

**Affiliations:** 1 Columbia University Irving Medical Center, 161 Fort Washington Ave, New York, NY 10032 USA; 2 Florida Atlantic University, Boca Raton, FL USA

**Keywords:** Mammography, Risk stratification, Breast cancer

## Abstract

**Background:**

U.S. professional organizations have provided conflicting recommendations on annual vs. biennial mammography screening. Potential harms of more frequent screening include increased anxiety and costs of false positive results, including unnecessary breast biopsies and overdiagnosis.

**Objective:**

To characterize current practices and beliefs surrounding mammography screening frequency and perspectives on using risk-based screening to inform screening intervals.

**Design:**

Semi-structured interviews informed by the Consolidated Framework for Implementation Research (CFIR).

**Participants:**

Patients, primary care providers (PCPs), third-party stakeholders (breast radiologists, radiology administrators, patient advocates).

**Main measures:**

Qualitative data, with a codebook developed based upon prespecified implementation science constructs.

**Key results:**

We interviewed 25 patients, 11 PCPs, and eight key stakeholders, including three radiologists, two radiology administrators, and three patient advocates. Most patients reported having annual mammograms, however, half believed having mammograms every two years was acceptable. Some women were worried early breast cancer would be missed if undergoing biennial screening. PCPs were equally split between recommending annual and biennial mammograms. Although PCPs were interested in using breast cancer risk models to inform screening decisions, concerns raised include time burden and lack of familiarity with breast cancer risk assessment tools. All breast radiologists believed patients should receive annual mammograms, while patient advocates and radiology administrators were split between annual vs. biennial. Radiologists were worried about missing breast cancer diagnoses when mammograms are not performed yearly. Patient advocates and radiology administrators were more open to biennial mammograms and utilizing risk-based screening.

**Conclusions:**

Uncertainty remains across stakeholder groups regarding appropriate mammogram screening intervals. Radiologists recommend annual mammography, whereas patients and PCPs were evenly split between annual vs. biennial screening, although both favored annual screening among higher-risk women. Breast cancer risk assessment tools may help facilitate decisions about screening intervals, but face barriers to widespread implementation in the primary care setting. These results will inform future implementation strategies to adopt risk-stratified breast cancer screening.

**Supplementary Information:**

The online version contains supplementary material available at 10.1186/s12885-022-09900-x.

## Background

Invitation to mammography screening has been associated with a 20% relative risk reduction in breast cancer mortality [1–3]. For decades, yearly mammograms were promoted, until 2009, when the U.S. Preventive Services Task Force (USPSTF) recommended biennial mammograms starting at age 50. Since then, there have been inconsistent recommendations on the frequency of mammogram screenings across professional societies. For example, the American College of Physicians also agree upon biennial mammograms [4]. However, the American College of Radiology (ACR) and the American College of Obstetricians and Gynecologists (ACOG) continue to recommend annual mammograms [5, 6]. Meanwhile the American Cancer Society recommends annual mammograms for women 45 to 54 years old but states that women over 55 can switch to biennial or continue annual mammograms based on shared decision-making between the patient and provider [7].

Although annual screening has enabled detection of cancers at earlier stages and improved breast cancer-specific mortality, over-screening presents unique harms including anxiety from abnormal mammograms, unnecessary biopsies for benign lesions, and increased use of surgical procedures with no clear mortality benefit [8, 9]. As a result, risk-based screening is receiving increasing attention [10]. Mammogram screening intervals can be tailored to a woman’s individual risk profile through the adoption of modeling to predict breast cancer risk. Although there are multiple validated models for predicting breast cancer risk, including the Breast Cancer Surveillance Consortium (BCSC) and Gail models [11, 12], patient and provider acceptance of determining mammogram frequency according to individual risk has not been assessed.

Our study objective is to understand current practices and beliefs surrounding mammogram screening practices among three stakeholder groups: patients, primary care providers (PCPs), and key stakeholders including breast radiologists, radiology administrators, and patient advocates. Additionally, we aim to characterize individual-level and structural barriers and facilitators to the adoption of risk-based mammography screening.

## Methods

### Study design

We conducted semi-structured interviews among women undergoing screening mammograms, PCPs, and other key stakeholders (breast radiologists, radiology administrators, and patient advocates) to explore their beliefs and behaviors surrounding mammogram screening frequency. This study was approved by the Columbia University Institutional Review Board.

### Recruitment and sample

We recruited patients, providers, and stakeholders who previously participated in related studies through email and phone. We interviewed a purposeful sample of patients who undergo screening mammography at our institution, aiming to capture perspectives of women from various socioeconomic and racial/ethnic backgrounds and different screening frequency. We interviewed a purposeful sample of providers who were primary care providers, aiming to capture perspectives from internists, family practitioners, and gynecologists. We interviewed a purposeful sample of key stakeholders who were not primary care providers who still had experience working with patients undergoing mammograms and interviewed breast radiologists, mammography center directors, or community/patient advocates.

### Data collection and analysis

Semi-structured interview guides for all three groups were based on prior literature and constructs adapted from the Consolidated Framework for Implementation Science (CFIR) [13]. One interview guide was developed for patients and a second interview guide was developed for providers and key stakeholders (Supplemental Tables 1 and 2). All participants were asked all questions in the interview guide and non-structured follow-up questions were utilized to clarify rationales for mammogram-related decision-making. Interviews were conducted with each participant individually using Zoom web-conferencing technology (Zoom Video Communications; San Jose, CA) between July 2020 and December 2020. The interviews were audio-recorded, transcribed verbatim and de-identified data was analyzed using the Dedoose software (SocioCultural Research Consultants; Manhattan Beach, CA). We used content analysis to systematically describe the meaning of the qualitative data [14, 15]. The coders used line-by-line coding and discussed code applications as a group to develop consensus. The analysis team members used the Dedoose training center to evaluate inter-rater reliability by generating a pooled-kappa coefficient to assess coding precision [16]. The final pooled-kappa score was (Pooled Cohen’s Kappa = 0.88), which indicated a high level of coding agreement among the coding team [17]. To ensure trustworthiness, direct quotations were provided to connect the results to the raw data.

## Results

A total of 25 patients, 11 providers, and eight key stakeholders participated in the semi-structured interviews (Table 1). Of the 11 providers, seven were internists, three were gynecologists, and one was a family practitioner. Of the eight key stakeholders, three were breast radiologists, two were radiology administrators, and three were patient advocates. The three patient advocates served multiple and various roles, including assisting patients with appointments, assisting patients with services such as housing and financial support, facilitating support groups for women diagnosed with breast cancer, and serving on committees that promote participation in clinical trials. We present the results in three main sections: patient perspectives, provider perspectives, and key stakeholder perspectives.

**Table 1:** Participant characteristics

**Patient Characteristics (*N*** **= 25)**	
Mean Age, **years (SD)**	56.2 (11.3)
Ethnicity, **N (%)**	
Hispanic/Latina	7 (28)
Non-Hispanic	18 (72)
Race, **N (%)**	
Asian	4 (16)
Black/African American	4 (16)
Hispanic	7 (28)
White	10 (40)
Primary Language, **N (%)**	
English	21 (84)
Spanish	4 (16)
**Provider Characteristics (*N*** **= 11)**	
Gender, **N (%)**	
Male	3 (27)
Female	8 (73)
Degree, **N (%)**	
MD	9 (82)
NP	2 (18)
Specialty, **N (%)**	
Internal Medicine	7 (64)
OB/GYN	3 (27)
Family Medicine	1 (9)
**Stakeholder Characteristics (*N*** **= 8)**	
Role, **N (%)**	
Radiologist (MD)	3 (38)
Radiology Administrator (RNs)	2 (25)
Patient Advocate	3 (38)

### Patient perspectives

#### Mammography screening frequency

Overall, most women (*n* = 19, 76%) reported having a mammogram every year. Of the women who reported yearly mammograms, most appeared to be motivated by their PCP recommendation and annual reminders.

*I tend to schedule my annual checkup like February/March timeframe … when my primary care physician puts that down there, I schedule an appointment and see whatever is available. [Patient #22].*

*I have been getting them every year for the most part for a number of years now. This year, I’m actually going to have one on [date], and I got a reminder last [month] that was, you know, about the year point. [Patient #7].*

Of the women who reported having a mammogram less frequently, two reported that their doctor recommended having a mammogram every two years instead of every year because their insurance wouldn’t cover it. Two women stated that they had forgotten their mammogram appointment and another woman stated that she is 77 years old and did not receive any more recommendations to have a mammogram.

Some women found the idea of biennial mammograms acceptable while others did not. Twelve women (48%) believed that having a mammogram every two years is acceptable if that was the recommendation from their healthcare provider. Another woman stated that the guidelines are confusing and wondered how to weigh differing recommendations. Several women believed that very high-risk women like those with a *BRCA1/2* gene mutation should be screened more often.

*I will also say that guidelines have changed several times in recent years that can be confusing, and this happens with other things as well. I think some years back there was information that women shouldn’t go every year, that it’s not necessary or that women can stop at a certain age or that women should start at a different age then what was previously recommended. So these things seem to change periodically which causes confusion and perhaps causes women to question the credibility. [Patient #7].*

*I think if you are in a high-risk category you should be surer to do your annual or what is recommended for your age or risk level. [Patient #9].*

Eleven women (44%) felt that two years is too long to wait to have a mammogram and they would feel more confident in having a mammogram every year, particularly if they are high-risk.

*I would personally not feel comfortable with that. […] Because I have been told and it’s been demonstrated to me that I am high risk. I am going through menopause and I just think that’s a long time to wait. I mean I have a kid, I don’t want to die in my 50s.*

#### Factors influencing mammography screening frequency

Participants named a variety of factors that influenced their decision to have a mammogram, including doctor’s recommendation (*n* = 14), reminder letters (*n* = 13), family history of breast cancer (*n* = 9), desire for early detection (*n* = 5), and age (*n* = 2). When asked “who orders your mammogram,” the majority reported their PCP or gynecologist. Annual reminder letters from radiology were another top factor that influenced a woman’s decision to have a mammogram. The majority (*n* = 17) received a letter as a reminder to have their annual mammogram, six received a phone call, and three reported receiving a reminder through the patient portal.

*It’s from the breast screening patient navigator at Columbia. I don’t know that this is my latest letter, but I know that I kept this letter as sort of sitting out just to remind me. Basically, it says if you have not had a mammogram in 1–2 years, we encourage you to call. [Patient #4].*

*Well usually I follow my doctor’s instructions. Since I started getting [the letters on] a certain day, I usually remind myself that the mammogram has to be done on that day and usually Columbia sends me a letter, a reminder, which is excellent, letting me know that my yearly mammogram is about to come up. [Patient #15].*

#### Perceptions of mammography benefits and harms

All participants believed that mammograms save lives and viewed the benefit of mammography as catching cancer in earlier stages and having the opportunity to intervene early. Some women (*n* = 13) viewed mammography as having harms such as radiation (*n* = 12), pain and discomfort (*n* = 4), and false positive results (*n* = 3).

*Early detection to see if there are any abnormalities; to see if anything else is wrong or right. The earlier the better, so there is a chance for early intervention if something is seen or found. [Patient #19].*

*I don’t even know if it’s radiation, but there is always talk about [it]. I think more it’s probably these false positives…undergoing procedures that just by being in a hospital are riskier in general. [Patient #4].*

### Provider perspectives

#### Mammography screening frequency

Among the 11 PCPs, six (55%) believed that women over 40 years old should receive yearly mammograms while the remaining five (45%) thought women should receive biennial mammography. All PCPs felt that their decisions were influenced by external guidelines, with five (45%) using USPSTF, three (27%) using ACOG, and one (9%) using ACR. Two only stated using “guidelines” without specifying further.

*Yeah, so, American College of OB/GYN still uses age 40 as the starting point for annual screening, so that’s what I have held to. [Provider #10].*

*I go by the Preventive Service Task Force Guidelines, so all things being equal without taking into account history of risk factors, family history risk factors or personal history risk factors, my baseline assumption is that we should be doing it every other year for women starting at 50 and stopping at 74. [Provider #8].*

Three PCPs felt that yearly reminders from breast radiology influence them to obtain yearly mammograms because patients request them. Eight (73%) providers said they incorporate patient preferences and concerns when deciding on frequency.

*I feel like those letters really drive my practice a lot because when the patient gets a letter from the hospital saying it’s time for your mammogram, I can’t possibly say, well I think it will be okay to wait another six months, you had one a year ago …but since they got it and they are requesting it, I’m sort of driven by that. [Provider #6].*

*In most cases patients are anxious for the annual and I can’t think of any patient who has requested less frequent mammograms. [Provider #2].*

Seven providers (64%) stated they screen patients more frequently when patients have a family history of breast cancer, and four (36%) screen patients more frequently when they have a history of abnormal mammogram.

*I honestly do not have a good mental algorithm for in whom that is most appropriate. The people who I usually aggressively promote more frequent intervals are those who have had abnormal mammograms in the past. [Provider #3].*

#### Communication with patients about mammograms

Most PCPs (*n* = 9, 82%) were confident about discussing mammograms with their patients; however, two (18%) providers felt they have knowledge gaps. When communicating breast cancer risks, five (45%) PCPs mainly discussed family history as a risk factor, three (27%) stated that they discuss all risk factors pertinent to patients’ histories, and three (27%) were hesitant about bringing up risk factors because it would incite unnecessary anxiety. Many PCPs stated that they do not discuss breast density with their patients, unless patients directly ask them, since they don’t know how density influences optimal management.

*I can talk to my patients about breast density all day, but the answer is, oh okay, so what should I do and I’m like, I don’t know. [Provider #3].*

In general, PCPs were reluctant to discuss harms surrounding mammograms. Five (45%) providers recommend mammograms to patients without any discussion about benefits and harms, while three (27%) only explain that benefits outweigh harms. Only one (9%) provider uses shared decision-making surrounding mammogram recommendations.

*I don’t usually say to the patient, “We could get a result and it could be abnormal and it might not be real and we may have to do other testing…” I don’t usually say that and maybe that’s wrong from an informed consent perspective, but I feel like if I tell them that, they are not going for the mammogram. Why would they go for a screening test that they don’t really want to go for, if I’m going to tell them that there is a potential harm? [Provider #6].*

#### Perceived benefits and harms of mammograms

All providers were strong proponents of mammogram screenings, with two (18%) stating there are no harms associated. All providers discussed some benefits of mammography screening, including early detection (*n* = 7, 64%), reduced likelihood of missing serious cancers (*n* = 4, 36%), reduced breast cancer mortality (*n* = 3, 27%), and reduced patient anxiety (*n* = 2, 18%).

*The goal with screening is to pick up on early cancers in order to prevent morbidity and mortality associated with breast cancer. I think it is often high on their healthcare maintenance to-do list and something that they want to do. [Provider #1].*

However, providers brought up harms, including unnecessary follow-up procedures (*n* = 5, 45%), increased patient anxiety (*n* = 3, 27%), and physical discomfort (*n* = 2, 18%). No provider felt that there was enough radiation emitted during exams to cause harm.

*The potential harms are over screening and over identification of breast lesions and changes that are going to lead to unnecessary worry and unnecessary procedures. [Provider #8].*

#### Barriers to risk-informed mammography screening frequency

Nine (82%) providers were interested in a calculated breast cancer risk for their patients. However, many felt there were too many barriers to utilizing risk scores in practice, such as time constraints during clinic visits (*n* = 5, 45%). Three (27%) PCPs stated it would have to be automatically calculated and accessible in the electronic health record (EHR) to be useful.

*I’m just thinking through a busy clinic visit where we have 15 minutes, sometimes less, and spending a significant chunk of that on calculating a risk for breast cancer. We end up doing it for statins, we end up doing it for aspirin, I guess if it already popped up and it was there and I didn’t have to do anything to plug numbers into a calculator, it would be a helpful part of a decision making. [Provider #1].*

### Stakeholder perspectives

#### Mammography screening frequency

Overall, six stakeholders (three radiologists, two patient advocates, and one radiology administrator) believed that women over 40 years old should receive yearly mammograms while the remaining two stakeholders (one patient advocate, one administrator) thought biennial mammography was acceptable for the general population. In particular, stakeholders endorsing yearly mammograms are influenced by prior experiences of seeing rapid progression of disease and seeing the negative impacts of delayed diagnosis. One stakeholder stated that social marketing campaigns also promote more frequent mammograms.

*It’s basically somewhat anecdotal in the sense that I have seen numerous times, as I’m 58 and I’ve been doing this a long time, what can happen if one doesn’t do it every year. In other words, a progression can happen very quickly. [Stakeholder #6, radiologist].*

*So I am comfortable, not at the first mammogram, but I am comfortable after 2 or 3 to explain that having a mammogram every two years would not be dangerous, but initially, again because of the population, I wouldn’t say every two years blanketly. [Stakeholder #2, administrator].*

*Without having clear ability to stratify risks, I think the goal of screening is to save as many people. I think the screening age 40 and doing it annually is at least well rounded and researched. [Stakeholder #1, radiologist].*

*The raw death numbers have not changed in decades. So, if the goal of this is to stop the deaths due to breast cancer, we haven’t really made much progress in that. So indolent disease or disease that is easily treated, even if it’s caught later. Early detection is not the end all and the be all and somehow that message is now cemented in our culture, early detection saves lives. No, it doesn’t. [Stakeholder #4, patient advocate].*

Although all three radiologists believe in annual screening, two radiologists state that they will defer the decision on frequency to the primary clinician and the patient’s decision. One administrator stated that her organization strongly promotes yearly mammograms, and that they send yearly screening reminders to patients. All three radiologists felt that patients of all ages should be screened at the same rate. However, two patient advocates and one administrator felt that younger and older patients should be screened less frequently.

*I think age, either on the low side or on the high side. I mean like continuing mammography in women that are 80 years old that never had a history of breast cancer, and then it comes down to what are you going to do for them at 80 years old. [Stakeholder #4, patient advocate].*

Two patient advocates, one administrator, and one radiologist stated that patients with a family history of breast cancer should be screened earlier and using supplemental methods.

*If they have any kind of genetic predisposition, a family member, if they have been tested in the past. Anything that will make them high risk, I think, yes, they should definitely also get yearly mammos. [Stakeholder #7, administrator].*

#### Communication with patients about mammograms

Three radiologists and one patient advocate stated they were confident in discussing mammograms with patients because it is their job to do so. Three stakeholders (one radiologist, one administrator, one patient advocate) tell patients that benefits of mammograms outweigh harms. One administrator does not explain any harms and only describes benefits to patients.

*I think a lot of our patients when they come, I think it’s sort of part of their routine. They understand that it’s an annual thing and so we don’t have a time carved out where we routinely speak with them, but certainly if they had questions. [Stakeholder #1, radiologist].*

All three radiologists and both administrators state that patients are informed about breast density through a letter. One radiologist, one administrator, and one patient advocate stated that higher density is described to increase breast cancer risk, while two radiologists state that ultrasounds are recommended for patients with higher breast density.

*When we do our community education, we also explain mammographic breast density and why it is considered putting woman at higher risk for breast cancer. [Stakeholder #2, administrator].*

*There is also now a statement about breast density so patients who have either heterogeneously dense or extremely dense breast tissue are required to be told as much and those patients are told that breast density can increase risk for breast cancer and that they should discuss with their provider the utility of adding some sort of supplemental screening test, although that’s where the language sort of stops. [Stakeholder #3, radiologist].*

#### Perceived benefits and harms of mammograms

 All three radiologists and one patient advocate mentioned that perceived benefits of mammograms include early detection and reducing mortality. Five stakeholders (two radiologists, two administrators, one patient advocate) stated that false positives and unnecessary procedures are the harms of mammograms. One patient advocate and one administrator felt that exposure to radiation was a harm and one patient advocate felt that mammograms heightened anxiety.

*Some of the other, softer side of things of negative consequences are false positive findings, [and] additional biopsies. [Stakeholder #1, radiologist].*

*There is a level of anxiety that occurs when you are called back to do additional film. Often times when you are called back, you’re not really told what it is they are seeing, what it is they are trying to rule out. You’re just told to come back and get the additional film. And so, I do think that there is an area where it causes a great deal of anxiety, there is a possibility that you might have to have several biopsies because of what is seen. [Stakeholder #8, patient advocate].*

#### Risk-informed mammography screening frequency

 Five stakeholders (one radiologist, two administrators, two patient advocates) said that breast cancer risk assessment tools could be well-received. However, all radiologists and one administrator were concerned about how accurately it can risk-stratify patients. One radiologist also stated that this type of tool would not be used if its recommendations go against radiologists’ screening philosophy.

*I would really appreciate it. I think it will be beneficial. Absolutely anything to help the community understand. [Stakeholder #7, administrator].*

*I think that’s a great idea, but I don’t think there is enough research data to accurately predict somebody’s risk. Certainly, there is a lot of thought about what is contributory, but if you think of a large population to see how that could be applied, I think there is a potential to miss a lot, especially if this or any risk model suggests that you are maybe at lower risk...We probably wouldn’t use it. [Stakeholder #1, radiologist].*

## Discussion

Our study showed diverse stakeholders, including patients, primary care providers (PCPs), radiologists, administrators, and patient advocates, hold divergent views about optimal mammogram screening frequency resulting in general uncertainty. For example, radiologists recommended annual mammograms, consistent with their society guidelines, whereas patients and PCPs were split between annual versus biennial screening, although both favored annual screening among women at higher risk. Breast cancer risk assessment tools may help facilitate decisions about screening intervals, but face barriers to widespread implementation in the primary care setting, including time burden, clinicians’ unfamiliarity with using risk calculators to guide cancer screening, and distrust of risk calculators’ accuracy.

One of the major factors influencing women’s decisions to pursue mammography was annual reminders to schedule a mammogram mailed by the radiology department. These reminders are universally applied to women following their initial mammogram, without attention to individual patient risk factors or preferences. Prior studies have shown that reminders are a highly effective means of influencing repeat use of mammography, and possibly more effective than education/motivation or counseling [18]. As a result, many patients may be requesting yearly mammograms during primary care appointments due to receipt of reminder letters.

Half of the patients interviewed favored annual screening because they were concerned the risks of biennial screening outweighed the benefits. Our interviews with PCPs and stakeholders show that patients are receiving incomplete information: we found that multiple stakeholders (e.g.*,* radiology, patient advocates, some PCPs) overemphasize the benefits of mammograms to patients, while minimizing potential harms of screening. For example, very few providers informed patients about the possibility of false-positive results, which have a cumulative probability of 61.3% among annual screeners compared to 41.6% for biennial screeners over a 10-year period [19, 20]. However, studies have shown significant consequences following a false-positive screening mammogram [21–23], including delaying subsequent mammograms [22] and experiencing significant distress (similar to that of being diagnosed with breast cancer) [23]. Economically, false-positive mammograms and breast cancer overdiagnoses cost an estimated $4 billion a year in the United States [24].

Despite the overemphasis on the benefits of annual screenings, many women express intent to follow their PCP’s recommendation about screening intervals. However, providers are faced with conflicting guidelines from ACOG and the USPSTF, as well as divergent patient preferences, when making recommendations to patients. Furthermore, our interviews revealed knowledge gaps among providers and patient advocates. For example, most providers used family history of breast cancer to determine whether patients are at high risk and most do not mention breast density. However, breast density is an established breast cancer risk factor [25]. Furthermore, three key stakeholders mentioned screening less frequently at extremes of ages although this has not been specifically recommended by professional organizations and has not been validated in prior studies. Breast cancer risk calculators can potentially enable providers to quickly integrate individualized risk factors in a standardized, evidence-based fashion which can guide their recommendations and facilitate a more nuanced discussion between patients and providers. They can also provide a way for increased education and awareness of other breast cancer risk factors beyond family history. However, despite their potential utility, barriers to the adoption of risk calculators persist, including time constraints, unfamiliarity with using them, and distrust of accuracy.

Strengths of our study include a wide set of stakeholders, ranging from racially diverse patients to physicians from multiple specialties to patient advocates, which captured more perspectives regarding mammogram frequency. Limitations of our study include that this was a single institution study conducted at an urban academic medical center so results may not be generalizable to community-based practice settings. Although we interviewed a diverse set of stakeholders, we had relatively few radiologists and patient advocates so responses may not be representatives of these subgroups. Although there were relatively few breast radiologists and patient advocates, because this is a single-institution study, we were able to interview a sizeable proportion of total breast radiologists and total patient advocates working within the department at our institution. Future studies should seek to further investigate the practices and beliefs of third-party stakeholders in this area beyond our single institution.

In conclusion, risk stratification tools for cancer screening are in early stages of development and require further research to maximize their clinical utility. As they become more widely adopted, other aspects of implementation and dissemination, particularly acceptability among providers and radiologists, need further investigation.

## Supplementary Information

**Additional file 1: Supplemental Table 1.** Interview guides for providers and key stakeholders. **Supplemental Table 2.** Interview guides for patients.

## Abbreviations

ACOG: American College of Obstetricians and Gynecologists
ACR: American College of Radiology
BCSC: Breast Cancer Surveillance Consortium
CFIR: Consolidated Framework for Implementation Science
EHR: Electronic health record
MD: Medical doctor
NP: Nurse practitioner
OB/GYN: Obstetrician/gynecologist
PCP: Primary care provider
RN: Registered nurse
SD: Standard deviation
USPSTF: U.S. Preventive Services Task Force
