# Supplementary material for: Patient, primary care provider, and stakeholder perspectives on mammography screening frequency: lessons learned from a qualitative study
Source: BMC Cancer. 2022 Jul 27;22:819. doi: 10.1186/s12885-022-09900-x (PMC9326136; doi:10.1186/s12885-022-09900-x)
Supplement: Supplementary file 1 — Additional file 1: Supplemental Table 1. Interview guides for providers and key stakeholders. Supplemental Table 2. Interview guides for patients. [file 12885_2022_9900_MOESM1_ESM.docx]

Supplemental table 1. Interview guides for providers and key stakeholders

**Thank you for meeting with me. I would like to start by asking for a brief summary about your healthcare/community setting, and how you are involved in decisions regarding the implementation of screening mammography programs and/or policies.**

**Next I would like to ask you a few questions about screening mammography**

- To start us off, can you please describe for me how mammography for breast cancer screening fits in your current practice/position within a healthcare/community setting?
- In your view, what are the benefits and harms of screening mammography?
  - - How important do you think these are?
- How often should women get screening mammograms and why?
  - What are your reasons for choosing this frequency?
  - Does this frequency depend on any factors?
    - If so, what are they?
  - Should some women receive mammography less often than once per year?
    - If so, what patient and organizational factors might make less frequent screening difficult to implement?
- What factors determine how often patients/clients at your institution or organization are referred for or receive a mammogram?
  - Are there any patient characteristics, including demographic, clinical, cultural, and socioeconomic?
  - Are there any organizational factors?
  - Are there any policy or other external factors, including payment structure?
- If applicable, does your practice or organization have any standard processes for scheduling patients for screening mammography?
  - Is there anything in the electronic health record?

**Breast Cancer Screening Communication:**

**I would now like to ask you a couple of questions about how your practice or organization communicates with women about breast cancer screening (if applicable).**

Provider-Level Self-Efficacy: *[breast radiologists]*

- Are you confident in your ability to discuss breast cancer screening with patients?
  - Are there any potential resources that would make discussing screening easier for you?
- In your practice, how frequently do you discuss breast cancer screening recommendations with patients?
  - What factors guide your recommendations?

Practice/Organization/Community-Level Processes:

- How does your practice/organization/community schedule and communicate the details of mammography appointments with patients?
  - Are patients sent reminders?
  - Is there anything about this communication that can be improved?
- How does your practice/organization/community communicate breast density results to patients?
  - Is there anything about this communication that can be improved?
- How does your practice/organization/community communicate the benefits and harms of mammography to patients?
  - Is there anything about this communication that can be improved?
- How does your practice/organization/community communicate breast cancer risk to patients?
  - Is there anything about this communication that can be improved?

**Intervention Implementation:**

**We are developing a web-based intervention that seeks to educate patients on breast cancer risk and breast cancer screening. The intervention will also calculate a patient’s personalized breast cancer risk and use this risk to guide shared decision making with patients about how often to undergo screening mammography.**

- How would you receive such an intervention in your practice/organization/community?
- How well would this intervention meet your patients’/clients’ needs?
  - Do you think that patients/clients would be willing to use the intervention?
- Does it sound like this intervention would be compatible with your clinical values and workflow?
  - How about those of your wider practice/organization/community setting?
  - Would the intervention replace or complement a current program or process?
- What factors do you think would make implementing such an intervention difficult?
- Are there any factors that might make implementing such an intervention easier?
- Whose support would we need to obtain to successfully implement such an intervention?

Supplemental table 2. Interview guides for patients

**Please describe your current breast cancer screening behaviors.**

Probes:

- How often do you currently receive a screening mammogram?
- How do you decide when to get a mammogram?
- Who orders your mammogram?
- Where do you receive your mammogram?
- Do you engage in any other types of breast cancer screenings? (e.g., breast self-exam, clinical breast exam, MRI, ultrasound)

**What factors influence your decision to have breast cancer screening?**

Probes:

- - Have you ever had an abnormal mammogram? Tell me more about that.
  - Have you ever had a breast biopsy? Tell me more about that.
  - What types of messaging or communications do you receive about screening mammograms?
    - Who do these communications come from?
    - Are these annual reminders?
    - How do they influence your screening decisions?
  - What breast cancer screening recommendations does your doctor give you?
    - How closely do you follow these recommendations?

**What barriers or issues, if any, do you encounter related to your breast cancer screening?**

Probes:

- Have you encountered any issues with your health insurance?
- Have you encountered any issues with accessing or getting your mammogram?
- Do you have any fears or concerns about having a mammogram?
- What are your cultural views about having a mammogram?
- How has COVID-19 affected you getting breast cancer screening?

**How do you perceive your risk of developing breast cancer compared to the average woman?**

Probe:

- Would you say that your risk is higher, lower, or the same?

**Do you think that a woman’s breast cancer risk should influence her mammogram screening schedule?**

Probe:

- Should some women get screened more often than others? (e.g., women with dense breast; black women who tend to get breast cancer at an earlier age)

**What do you know about the benefits of a mammogram?**

Probes:

- Do you think that mammograms save lives?
- What types of cancers do you think that a mammogram can detect? (e.g., invasive or deadly cancers)

**What do you know about the harms of a mammogram?**

Probes:

- Do you think that too much screening is harmful?
- How do you feel about having a mammogram every 2 years?

**How do you perceive your risk of developing breast cancer compared to the average woman?**

Probe:

- Would you say that your risk is higher, lower, or the same?

**Do you think that a woman’s breast cancer risk should influence her mammogram screening schedule?**

Probe:

- Should some women get screened more often than others? (e.g., women with dense breast; black women who tend to get breast cancer at an earlier age)

**What do you know about the benefits of a mammogram?**

Probes:

- Do you think that mammograms save lives?
- What types of cancers do you think that a mammogram can detect? (e.g., invasive or deadly cancers)

**What do you know about the harms of a mammogram?**

Probes:

- Do you think that too much screening is harmful?
- How do you feel about having a mammogram every 2 years?

**We are developing a web-based intervention that seeks to educate women about their breast cancer risk and breast cancer screening. Do you think that learning more about the benefits and harms of screening mammograms would influence your screening decisions?**

**Do you think that learning more about your personal breast cancer risk would influence your screening decisions?**

**What should an ideal intervention about breast cancer screening include?**

Probes:

- Would you be interested in learning more about breast cancer risk factors?
- Would you be interested in learning more about what it means to have dense breasts?
- Would you be interested in learning more about the types of mammograms (e.g., 3D vs. 2D)
- What else are you interested in learning about breast cancer screening?
